# Cutting Performance of Randomly Distributed Active Abrasive Grains in Gear Honing Process

**DOI:** 10.3390/mi12091119

**Published:** 2021-09-17

**Authors:** Yang Gao, Fuwei Wang, Yuan Liang, Jiang Han, Jie Su, Yu Tong, Lin Liu

**Affiliations:** 1 Institute of CIMS, Hefei University of Technology, Hefei 230009, China; 2008034@nmu.edu.cn (Y.G.); nuaawfw@163.com (F.W.); 2 College of Mechatronic Engineering, North Minzu University, Yinchuan 750021, China; ly18895087502@163.com (Y.L.); ty932683028@163.com (Y.T.); 3 School of Mechanical Engineering, Ningxia University, Yinchuan 750021, China; sujie9493@163.com; 4 Department of Mechanical Engineering, University of Kansas, Lawrence, KS 66045, USA; linliu@ku.edu

**Keywords:** power gear honing, active abrasive grain, distribution state, honing performance

## Abstract

In power gear honing, the random distribution of abrasive grains on the tooth surface of the honing wheel is the main factor that influences the machining performance of high-quality hardened gears. In order to investigate the micro-edge cutting performance of the active abrasive grains on the workpiece gear, the real honing process is simplified into a micro-edge cutting model with random distribution of active abrasive grains in the cells of the meshing area by obtaining the random distribution states such as the position, orientation and quantity of the honing wheel teeth. The results show that although the active abrasive grains are distributed at different locations, they all experience three types of material removal—slip rubbing, plowing and cutting—allowing the gear honing process to have the combined machining characteristics of grinding, lapping and polishing. The active abrasive grains in first contact produce high honing force, high material removal efficiency and poor surface roughness on the machined workpiece, while the latter ones have the opposite effects. The dislocation angle affects the chip shape and chip discharging direction, and the highest honing force and material removal efficiency is achieved with a dislocation angle of 135°. The higher the number of active abrasive grains in a given contact area, the higher the material removal efficiency.

## 1. Introduction

Precision hardened gears are widely used and in great demand in fields such as high-performance automobiles and high-end robots, which have high requirements for the lifespan, transmission efficiency, and load capacity of gears. Power gear honing is a new technology that offers an alternative to grinding for precision-hardened gears, which has the advantages of improving the geometric accuracy of tooth orientation and tooth shape, prolonging the working life of gears, reducing transmission noise and increasing the bearing capacity of tooth surfaces [1,2,3,4]. However, gear honing is a complex multi-grain micro-edge cutting process with a large number of irregularly distributed abrasive grains on the tooth surface of the honing wheel [5], removing the machining allowance [6] and generating the typical honing grooves [2,6] along the contact traces [7] in the contact area with a smaller honing force [8] and lower honing speed [9]. Hence, it is much more complicated than ordinary turning, milling, grinding and other machining methods, restricting the wide application of honing technology.

Active abrasive grains play a key role in determining the machining performance of high-quality hardened gears [10,11]. Micro-edge honing with randomly distributed active abrasive grains is able to modify the tooth shape and tooth orientation deformation after quenching, improve the geometric accuracy of gears, increase the tooth surface overlap, improve the tooth surface loading capacity, and produce regular honing patterns on the workpiece tooth surface, and is thus capable of effectively reducing gear transmission noise [12]. The honed grooves can store lubricant, thus reducing frictional resistance and increasing gear life [1,4]. The abrasive grain applies a high level of contact pressure to the workpiece, inducing compressive stress on its tooth surfaces. The compressive stress improves the wear resistance of honed workpiece surface and thereby increases the working life of gears [13]. Therefore, it is essential to obtain the random distribution state of abrasive grains of honing wheels for controllable precision and ultra-precision honing.

The distribution and shape of abrasive grains on the tooth surface of honing wheels can be detected by experimental methods such as the stylus profilometer method, laser-focusing method, laser triangulation method, microscope-focusing method and light interference method. In order to reconstruct the 3D micro-topography of the tooth surface of honing wheels, one approach is to consider that the abrasive grains are in a normal distribution and establish the topography of the tooth surface of honing wheels by a probability density function [14]. Another approach is to simulate the microstructural evolution of cubic boron nitride (CBN) coating on the tooth surface by the magnetron sputtering method based on phase field theory to obtain the micro-topography of abrasive grains [10]. However, in practice, the interaction between the abrasive grains and the workpiece tooth surface is so complex that it is difficult to observe the micro-cutting behavior of abrasive grains in the contact zone at different times.

In order to simplify the mechanism of interaction between the abrasive grain and workpiece in honing, researchers often use the grinding principle to explain the honing process [15,16]. E. Brinksmeier [6] simplified this to low-speed grinding and designed a low-speed grinding experimental device to perform grinding tests at 0.3–3 m/s, finding that the rolling and sliding motion of hard abrasive grains between the workpiece and the tool removes material from the surface of the workpiece. To further reveal that honing is a typical form of low-speed grinding, Lv [17] designed a low-speed scratching test with a single abrasive grain, and the results showed that the arc-shaped chips are similar to the wedge-shaped chips of low-speed grinding, and whether the abrasive grain is in the state of micro-plowing or micro-cutting mainly depends on the scratching speed and depth. Furthermore, E. Brinksmeier [18] pointed out that the chip formation in the honing process can be equivalent to the chip formation mechanism of low-speed grinding.

Thus, the honing process can be considered as a low-speed multi-grain grinding process. In multi-grain grinding, Guo [19] believed that the extrusion height of abrasive grains is randomly distributed, and only a few active abrasive grains play a cutting role in grinding. Hence, the surface quality and machining efficiency of precision and ultra-precision machining of hard and brittle materials can be improved by controlling the 3D morphology and extrusion shape of active abrasive grains as well as the selection of small cutting depths [20]. According to Xie [21], the greater the extrusion height, extrusion volume, stiffness and front angle, and the smaller the back angle, main deviation angle and secondary deviation angle, the better the cutting performance of the grinding wheel. Therefore, the geometric characteristics of active abrasive grains on the surface of grinding wheels play an important role in the removal of workpiece materials, grinding force, surface roughness, machining efficiency and surface quality, and the grinding mechanism of active abrasive grains offers valuable insights into the machining characteristics of gear honing [22,23,24,25].

At present, it is still challenging to study the influence of parameters such as the distribution state, geometric characteristics and the number of CBN abrasive grains on honing performance. This is because the abrasive grains are randomly distributed on the honing wheel, and the geometry is irregular and the spatial meshing motion is complicated, which makes the micro-cutting process between the abrasive grains and the workpiece more complex [1,4].

In this paper, an equivalent multi-grain grinding model is first proposed to represent the material removal process of randomly distributed active abrasive grains in the gear-honing process. Then, the honing wheel tooth surface is divided into a certain number of regular cells, and the randomly distributed active CBN abrasive grains on the cells are used to study the randomness of distribution position and the uncertainty of geometric characteristics of the abrasive grains on the honing wheel tooth surface. Subsequently, the microscopic morphology of CBN abrasive grains in a unit cell on the tooth surface of honing wheel is simulated based on the phase field theory, and the geometric and positional information, such as the size, shape, orientation and distribution of the active abrasive grains in the cell, is extracted. Finally, the finite-element method is applied to simulate the process of removing the workpiece material by multi-abrasive grains along the contact traces, and the material removal performances, such as material removal rate, chip breakage and surface roughness influenced by active abrasive grains, are explored and discussed.

## 2. Modeling of Multi-Grain Micro-Edge Honing Process

**Figure 1:**
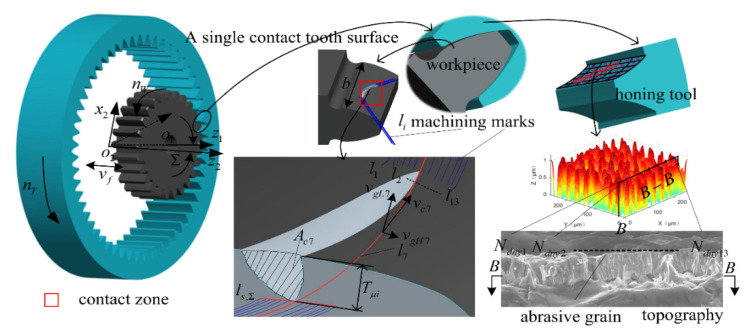
Honing mechanism of multi-abrasive micro-edge honing.

As shown in Figure 1, computer numerical control (CNC) internal power honing is a forced meshing motion between the workpiece gear and the honing wheel. During the honing process, the heat-treated workpiece gears are clamped onto the CNC honing machine and produce a certain axis intersection angle with the precision honing wheel, thus forming a forced meshing state with intersecting axes [10,26]. The CBN grains on the tooth surface of the honing wheel significantly interfere with the workpiece tooth surface in the engagement area. As the hardness of the CBN grains deposited on the tooth surface of the honing wheel is much higher than the hardness of the tooth surface to be machined, the dynamic active grains ($N_{dyni}$) are pressed into the tooth surface of the workpiece under a high contact pressure ($F_{ni}$). In order to complete the processing of workpiece with tooth width b, the active abrasive grains and the workpiece rotate at a set speed around their respective rotary center-lines under the precise control of the electronic gearbox of the CNC system, and the honing wheel causes a reciprocal feeding motion at a speed $\left(v_{f}\right)$ along its axial direction. At this moment, the active abrasive grains press into the tooth surface of the workpiece slides against the tooth surface of the workpiece at a low cutting speed (0.2~16 m/s) along the contact line ($l_{i}$). The cutting depth of the abrasive to the workpiece starts from zero, gradually increases to the maximum, and then cuts out to zero. When the cutting depth is very small, the force between the abrasive grain and the workpiece is small, the material undergoes elastic deformation, and the abrasive grain slips through the tooth surface of the workpiece. When the depth of the cut reaches a certain value, the contact stress between the abrasive grain and the workpiece reaches the yield limit of the material, and then the material undergoes plastic flow and accumulates on both sides of the abrasive scratch. When the contact stress is greater than the material fracture strength, the material fractures and develops into chips, thereby completing the machining of the workpiece. The active abrasive grains produce an arc texture that is typical of honing, and remove a layer of chips with the maximum cross-sectional area $\left(A_{ci}\right)$ and maximum depth of cut ($T_{\mu i}$) on the workpiece tooth surface; thus, the finishing of hardened gears is achieved.

Based on the above analysis, it is clear that the basic principle of material removal in power gear honing relies on the reciprocal low-speed cutting between the active abrasive grains on the tooth surface of the tool and the tooth surface of the workpiece, which has the typical characteristics of a low-speed grinding process. Only when the active abrasive grains in the contact area reach a certain cutting depth, where the cutting stress is greater than the material fracture strength, can the material on the workpiece tooth surface be removed, which is essentially the low-speed micro-edge grinding of the workpiece material by randomly distributed active abrasive grains.

## 3. Simulation and Experimental Verification

### 3.1. Modeling of Randomly Distributed Active Abrasive Grains

**Figure 2:**
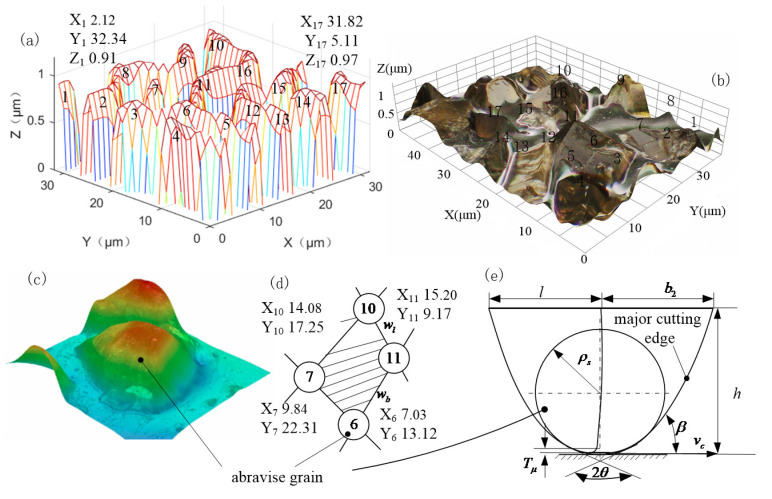
Modeling of randomly distributed active abrasive grain. (**a**) MATLAB simulation of microscopic geometry and distribution of CBN abrasive grains; (**b**) Microscopic geometry and distribution of CBN abrasive grains observed by digital microscope; (**c**) Microscopic geometry of single CBN abrasive grain; (**d**) Modeling of the space of active abrasive grains; (**e**) modeling of the geometry of single CBN grain.

Based on the theoretical model of the generation of CBN coating micro-morphology, the CBN coating on the tooth surface of the honing wheel was designed and prepared, and the information on the distribution and number of abrasive grains was extracted for multi-abrasive micro-edge cutting simulation and experimental verification. Specifically, after dividing the steel substrate honing wheel tooth surface into cells, take any one cell as the reaction space. The phase field method was used to simulate the process of grain nucleation, coarsening and final film formation during the microstructure evolution of CBN abrasive grains generated by magnetron sputtering. The microscopic geometry and distribution state of the CBN abrasive grains (Figure 2a) were obtained by applying MATLAB R2016 software. When the magnetron sputtering preparation process parameters such as temperature, time, Ar flow rate, *N*_2_ flow rate, and power are changed, the number $N_{d}$, spacing $w_{l}\times w_{b}$, orientation angle β, geometry, and position of the effective abrasive grains in the cell vary as well. By obtaining the optimal preparation process parameters, the CBN coating was prepared on the steel substrate by applying the SP-6A magnetron sputtering table. The distribution state of CBN abrasive grains was measured using a digital microscope Hirox HRX-01 and a turret-type motorized zoom lens HR-500E, as shown in Figure 2b, and the geometry of a single abrasive grain is shown in Figure 2c.

The spacing of the active abrasive grains was derived from the coordinate values in Figure 2d. The single CBN grain in Figure 2c was equivalently simplified to the geometry shown in Figure 2e, and the six main parameters of length *l*, width *b*_2_, height *h*, tip radius $\rho_{s}$, tip angle 2θ, and orientation angle β were used to control the geometry and orientation of the grain to construct a randomly distributed active grain model for multi-grain micro-edge cutting simulation.

### 3.2. Finite Element Simulation

As shown in Figure 1, the complex spatial meshing motion between the honing wheel and the workpiece was equivalently transformed into the honing motion of the active abrasive grains randomly distributed in the cells of the honing wheel tooth surface along the machining traces *l_i_*. Considering that honing process has the characteristics of a low honing speed, small honing force, small depth of cut, no tooth surface burn, small volume of abrasive grains and high hardness, the following equivalence is made for multi-abrasive micro-edge honing teeth: (1) After dressing the abrasive grains with a diamond pen before honing, the geometry of CBN abrasive grains is more consistent. (2) As the length of contact traces between active abrasive grains and the workpiece is short, the honing force is small, the honing speed is low, the hardness of the abrasive grains is much higher than the hardness of the workpiece material, and the wear of the abrasive grains is extremely small, so the effect of abrasive grain wear, fracture and shedding on the material removal rate is ignored and the CBN abrasive grains are treated as rigid bodies. (3) As the honing speed is very low compared with grinding, the volume of abrasive grains and the depth of cut are small, no thermal stress damage will occur on the tooth surface of the workpiece, so the effect of cutting heat on machining accuracy, metallographic organization and other chemical changes is ignored. (4) The material of the machined workpiece tooth surface is isotropic. (5) The influence of the vibration of abrasive grain and workpiece on the cutting process is ignored.

In actual processing, each randomly distributed active abrasive grain is in point contact with the workpiece for independent dynamic honing, so the process parameters are set by the random threshold method in order to truly reflect the active abrasive grain micro-edge honing characteristics, as shown in Table 1.

The simulation was performed using ABAQUS2016. The size of workpiece was 50 µm × 30 µm × 6 µm, considering the calculation efficiency and feasibility. The Johnson–Cook plastic model was adopted during simulation. Based on references [27,28], the material parameters for workpiece and CBN abrasive grain were obtained experimentally, as shown in Table 2.

20CrMnTi is a hard plastic material with high hardness on the tooth surface and good plastic toughness inside after heat treatment. The J–C shear failure criterion was adopted as the damage criterion for the workpiece material. The equivalent plastic strain is applied to define the damage parameters based on the element integration point, and can be expressed as:

(1) $$\delta =\overset{n}{\underset{j=1}{\sum }}\left(\frac{\Delta {\bar{\xi }}^{pl}}{{\bar{\xi }}_{D}^{pl}}\right)$$

When δ≥1, the element begins to fail. In Equation (1), $\Delta {\bar{\xi }}^{pl}$ is the equivalent plastic strain increment at the integration point of the element, and ${\bar{\xi }}_{D}^{pl}$ is the critical equivalent plastic strain. ${\bar{\xi }}_{D}^{pl}$ is obtained from the J-C shear failure criterion, and is expressed as:

(2) $${\bar{\xi }}_{D}^{pl}=\left[D_{1}+D_{2}exp\left(D_{3}\frac{\sigma_{p}}{\sigma_{Mises}}\right)\right]\times \left[1+D_{4}In\left(\frac{\dot{\bar{\xi }}}{\dot{\bar{\xi_{0}}}}\right)\right]\times \left[1+D_{5}\left(\frac{T-T_{room}}{T_{melt}-T_{room}}\right)\right]$$

where $\sigma_{p}$ and $\sigma_{Mises}$ are the hydrostatic stress and the von Mises stress, $\dot{\bar{\xi_{0}}}$ and $\dot{\bar{\xi }}$ are quasi-static compression test strain rate and equivalent plastic strain rate, *T*, $T_{room}$ and $T_{melt}$ denote the cutting temperature, room temperature and melting temperature, respectively. The J–C damage parameters of 20CrMnTi are shown in Table 3. The analysis adopted the dynamic display algorithm, and the analysis step was set as 2.5 $\times 10^{-5}s$ according to the amount of abrasive feed in the cutting layer area. Since each abrasive grain is in contact with the cutting layer independently, the contact form is chosen to be face-to-face during calculations.

### 3.3. Comparison of Simulation and Experiment Results

#### 3.3.1. Microscopic Morphology

**Figure 3:**
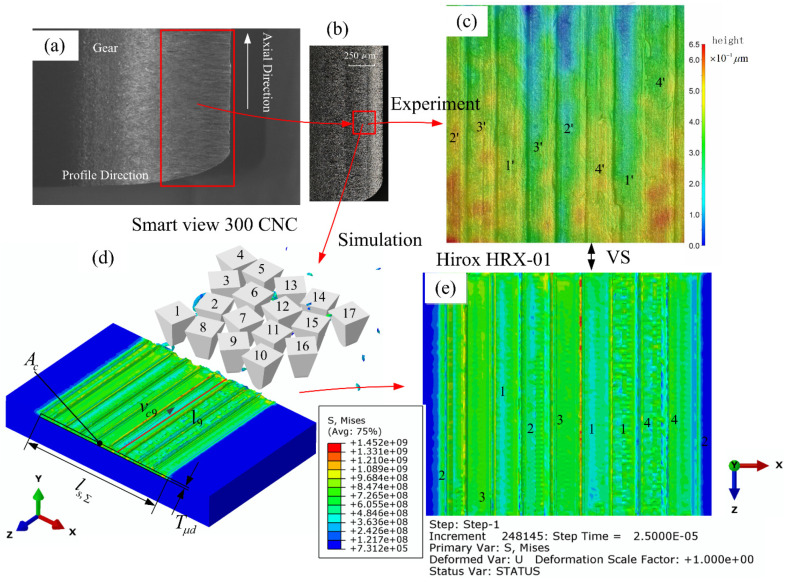
Comparison of simulation and experiment results of multi-grain honing process. (**a**) The micromorphology of the tooth surface of the workpiece under Smart View 300 CNC; (**b**) Micro-geometry of single-tooth local zoom under Hirox HRX-01; (**c**) A local enlarged view of the (**b**); (**d**) Finite element simulation results; (**e**) Top view of (**d**).

The simulation and experiment parameters are shown in Table 4. After the simulation and experiment of multi-grain honing, the micromorphology of the tooth surface of the workpiece is shown in Figure 1. The specific method is shown in Figure 3a. After the honing experiment, one tooth of the workpiece was wire-cut with GF CUT C350 EDM, and the Smart View 300 CNC profile imager was used to measure the micromorphology of the tooth surface. Hirox HRX-01 was applied to observe the micro-geometry of the workpiece from smaller size and different viewpoints, as shown in Figure 3b,c. The finite element simulation model was established based on the phase field method shown in Figure 2, and the simulation results are shown in Figure 3d,e.

Comparing the grooves marked 1’, 2’, 3’, 4’ in Figure 3c and the grooves marked 1, 2, 3, 4 in Figure 3e, the experimental results can effectively support the simulation results. The simulated biggest depth of cut was 0.02 µm smaller than the experimental one, and the difference between the simulated and experimental contact traces was 1.33µm in length. The simulated effective contact length is 1.02 µm greater than the experimental one, and the simulated honing area is smaller than the experimental one by 0.15 µm^2^. The error is less than the effect of single abrasive grain, and the reliability of the finite-element simulation results is acceptable.

**Figure 4:**
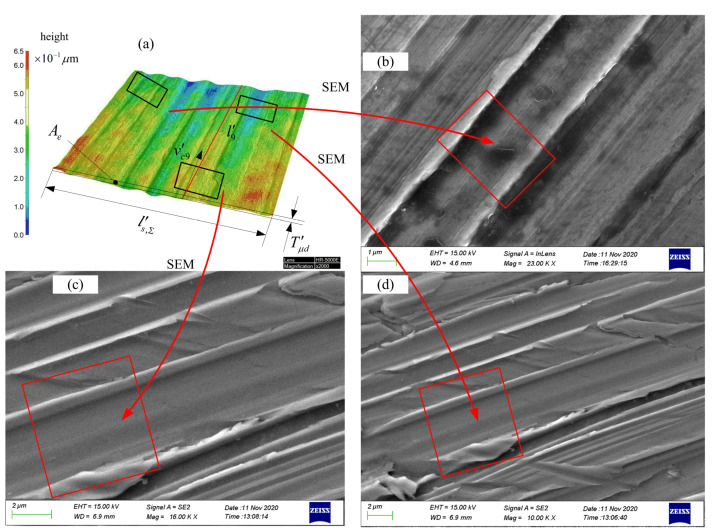
Partial enlargement of experimental results. (**a**) Local enlarged view of Figure 3b; (**b**–**d**) Local enlarged views of (**a**).

In order to observe the multi-grain cutting process at a more microscopic size, three positions were selected in Figure 4a, and the morphology of the workpiece surface after cutting by randomly distributed abrasive grains was observed and measured using the Zeiss SIGMA 500 thermal field-emission scanning electron microscope, as shown in Figure 4b–d. The micromorphology of the tooth surface of the workpiece after honing has also been experimentally proven in the literature [29]. The surface grooves of the workpiece are much closer to the finite-element calculation results, indicating that the simulation method used in Section 3.2 is feasible.

#### 3.3.2. Honing Force

To further verify the reliability of the finite-element simulation results, we used a flat face grinding wheel as an equivalent to the honing wheel and an external workpiece as an equivalent to the workpiece gear, based on E. Brinksmeier [6]. The honing speed $v_{c}$ at an arbitrary point can be obtained by applying the equivalent model for grinding the outer circle of the workpiece with a flat face grinding wheel. Therefore, the experimental honing force can be measured by equivalently transforming the honing process into a low-speed surface grinding process with $v_{c}$ = 0.1–16 m/s.

**Figure 5:**
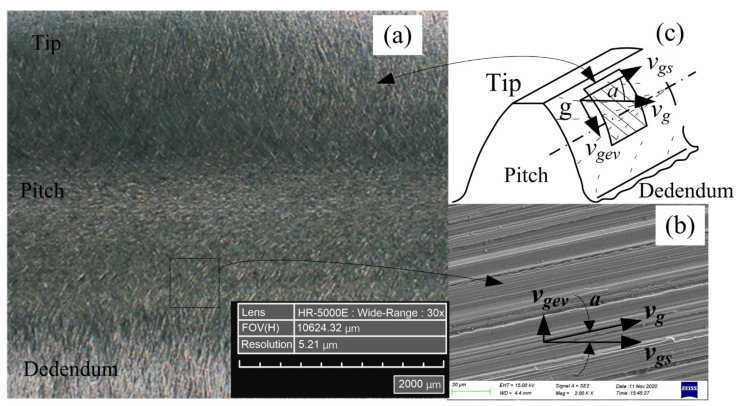
Modeling of sliding velocity of abrasive grains on tooth surface. (**a**) Honing texture of single tooth surface; (**b**) Honing speed decomposition; (**c**) Local enlarged view of (**a**).

The honing process was carried out with the multi-grain micro-edge honing principle shown in Figure 1, and the micromorphology of the tooth surface after honing is shown in Figure 5. The relative sliding velocity $v_{g}$ of the abrasive grains at point g on the tooth surface trace $l_{i}$ is decomposed into the rolling velocity $v_{gev}$ and the spiral velocity $v_{gs}$, where $v_{gev}$ denotes the the rolling velocity between the honing wheel and the workpiece along the tooth height direction of the effective contact trace, and $v_{gs}$ represents the spiral velocity between the honing wheel and the workpiece along the tooth width direction of the effective contact trace. The superposition of these two directional velocities produces the unique honing pattern shown in Figure 5a on the tooth surface of the workpiece. The relative motion of the honing wheel and the workpiece is responsible for the formation of the typical honing pattern on the tooth surface of the workpiece. The sliding speed $v_{g}$ can be expressed as:

(3) $$v_{g}=v_{gs}cosa=v_{gev}sina$$

where a is the angle between $v_{g}$ and $v_{gev}$. According to E. Brinksmeier [6], honing speed $v_{c}$ can be divided into longitudinal sliding speed $v_{ct}$ along the tooth profile direction and transverse sliding speed $v_{ca}$ along the tooth width direction. The tangential direction speed $v_{ct}$ and axial speed $v_{ca}$ in flat face external grinding can equivalently replace the rolling speed $v_{gev}$ and spiral speed $v_{gs}$ in honing, so that it can be unified with the honing speed of the workpiece tooth surface in Figure 5. Thus, the honing speed $v_{c}$ can also be calculated from the vector sum of the longitudinal sliding speed $v_{gev}$ along the tooth profile direction and the transverse sliding speed $v_{gs}$ along the tooth width direction, which reads:

(4) $$v_{c}=\sqrt{v_{gev}{}^{2}+v_{gs}{}^{2}}$$

By controlling the longitudinal sliding speed along the tooth profile and the transverse sliding speed along the tooth width, the honing force corresponding to that honing speed can be equivalently measured.

**Figure 6:**
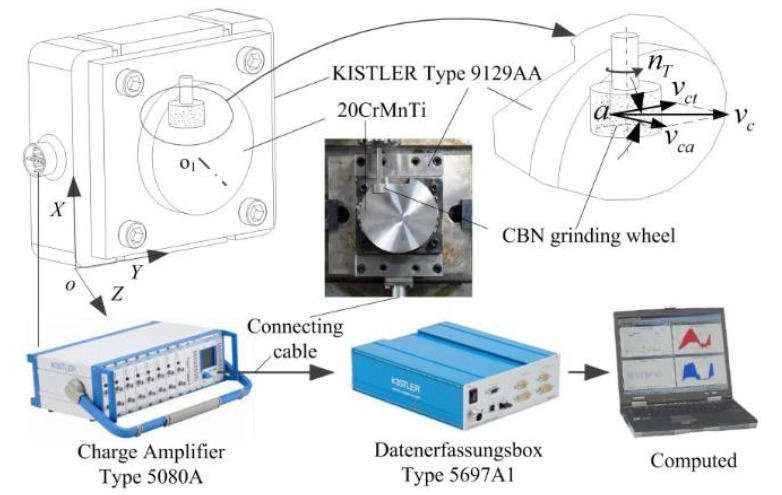
Experimental system of honing force for multi-grain micro-edge honing.

According to the above equivalent method of honing processing, the honing force testing system was established, as shown in Figure 6. The force sensor KISTLER 9129AA and the 20CrMnT workpiece were mounted on the table of MAZAK VARIAXIS j-500 machining center, and the CBN tool was clamped on the machine spindle for the active abrasive micro-edge honing experiment.

The honing force is calculated by coupling the average honing force of a single abrasive grain ${\bar{F}}_{ng}$, the number of active abrasive grains $N_{act}$, the number of dynamic abrasive grains $N_{dyn}$, the effective cutting width $b_{1}$ and the effective contact length $l_{e,\Sigma }$, which is expressed as:

(5) $$F_{n}={\bar{F}}_{ng}N_{act}={\bar{F}}_{ng}N_{dyg}l_{e,\Sigma }b_{1}$$

**Figure 7:**
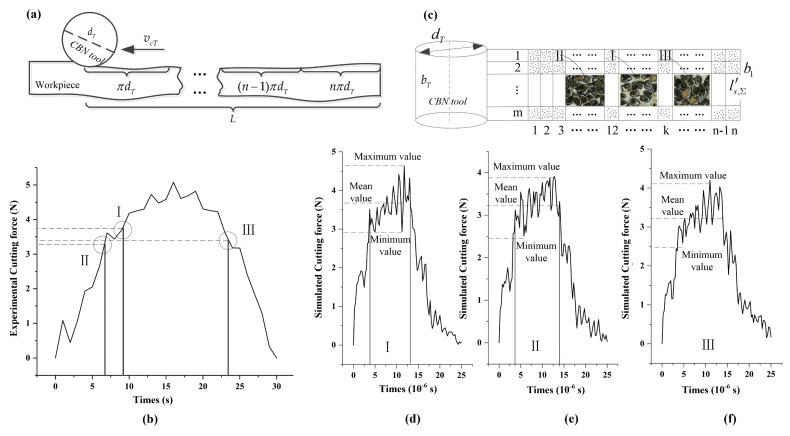
Cutting force between simulation and experiment of multi-milling process. (**a**) Mechanism of equivalent honing processing; (**b**) Honing force for 1 grinding cycle in 30 s; (**c**) Unfold the cylindrical honing wheel into m×n rectangular cells; (**d**–**f**) The total honing forces of the engagement areas I, II and III.

Based on the equivalent honing processing mechanism shown in Figure 7a, the number of active abrasive grains involved in chip formation in the meshing area of the workpiece gear and honing wheel was experimentally counted, and the average honing force of a single abrasive grain was calculated. The honing force for 1 grinding cycle in 30 s was collected, as shown in Figure 7b. The cylindrical honing wheel is graphically unfolded as a rectangle and divided into m×n rectangular cells, each with the length and width dimension $l_{s,\Sigma }^{\prime }\times b_{1}$, as shown in Figure 7c. Hence, the honing force of a cell can be derived by extracting the information of abrasive grains in cell $I_{1\times 12}$, ${\mathrm{II}}_{1\times 3},\;{\mathrm{III}}_{1\times k}$ and establishing an active abrasive micro-edge cutting model. Summing the honing force of the 3rd, 12th and kth column and mth row cells, the total honing forces of the engagement areas I, II and III were obtained, as shown in Figure 7d–f. The simulated and experimentally measured honing forces in each engagement zone generally follow the same trend, i.e., they increased from zero and then gradually decreased to zero after a period of stable cutting. Furthermore, by quantitatively comparing the average honing force magnitude in each tiny time period of the engagement zones I, II and III, it was found that the relative errors between the theoretical and experimental values were 3.69%, 2.19% and 1.39%, respectively, indicating that the simulation and the experiment results were nearly the same. Therefore, the simulation approach can be used as an alternative to the experiment when studying the micro-edge honing processing characteristics of randomly distributed active abrasive grains.

## 4. Results and Discussion

### 4.1. Effect of Abrasive Grain Distribution on Honing Process

**Figure 8:**
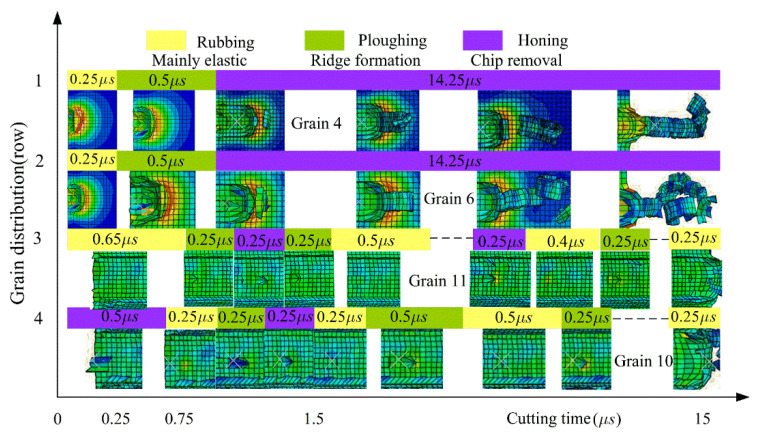
Distribution of abrasive gains and honing status.

**Figure 9:**
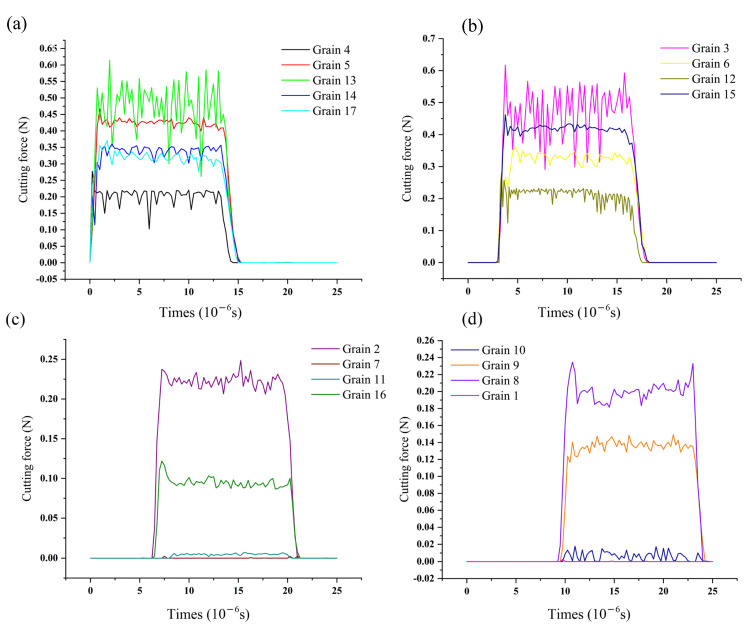
Honing force of single abrasive grains distributed in different rows. (**a**) The first row; (**b**) The second row; (**c**) The third row; (**d**) The fourth row.

According to the process parameters in Figure 3, the honing processes of abrasive grains 4, 6, 11 and 10, distributed in different rows and columns, were investigated. The variation rules of material removal pattern, material removal rate, surface roughness and honing force were analyzed according to the surface morphology and chips of the workpiece after honing, as shown in Figure 8 and Figure 9.

As shown in Figure 8, the abrasive grain 4 in the first row of slides against the workpiece material and generated an elasto-plastic deformation with a duration of 0.25 μs, after which the abrasive grain started plowing, leading to the bulging of the workpiece material on both sides and generating a small amount of tiny abrasive chips with a duration of 0.5 μs. After that, the abrasive grain entered the micro-edge honing stage, with a large number of long bar-shaped chips being generated and fractured, until the removal of the interfering workpiece material is completed on the entire contact trail. This showed that the abrasive grains 4, 5, 13, 14, 17 located in the first row performed three types of material removal on the workpiece material—slip rubbing, ploughing and honing—with honing taking longer than the other two stages. During the whole material removal process, the values of honing force, material removal rate and surface roughness were gradually increased from minimum to maximum. The abrasive grains in row 2 displayed similar material removal characteristics to the grains in row 1. However, when the abrasive grains in rows 3 and 4 were in contact with the workpiece, they were constantly changing between slip rubbing, ploughing and honing, and the slip rubbing time and ploughing time were obviously greater than the honing time.

It was found that the honing processes of abrasive grains distributed in different locations would experience three types of material removal: sliding, ploughing and cutting. The abrasive grains located in rows 1 and 2 were mainly cutting, which is similar to grinding; the abrasive grains in rows 3 and 4 were mainly material removal by slip rubbing and ploughing, and mainly served as polishing and grinding for the workpiece.

The cutting force for single abrasive grain is shown in Figure 9, substituting into Equation (2) and the relationship between the cutting forces of different rows of abrasive grains can be derived: $F_{n1}>F_{n2}>F_{n3}>F_{n4}$. It is evident that the abrasive grains in rows 1 and 2 produced high honing forces, high material-removal efficiency and poor surface roughness of the workpiece, while the abrasive grains in rows 3 and 4 showed the opposite results.

The main reason for the above pattern is the random distribution characteristics of the abrasive grains. The abrasive grains are spaced at different intervals and in different positions, so the latter grains are only able to remove the material that was left by the former grains. As the 1st and 2nd row of abrasive grains remove most of the interfering material, the material that can be removed by the 3rd and 4th row of abrasive grains is greatly reduced, at which point the rubbing and ploughing time between the abrasive grains and the workpiece significantly increases compared to the time of the first two rows. Here, the honing effect of the abrasive grains is no longer obvious, and is primarily a grinding and polishing effect, showing a sharp reduction in honing force, a lower material removal rate, fine honing grain grooves and better surface roughness. Therefore, the random distribution of active abrasive grains affects the material removal pattern, material removal rate, surface roughness and honing force, displaying has a combined effect of grinding, lapping and polishing.

### 4.2. Effect of Orientation Angle on Honing Process

**Figure 10:**
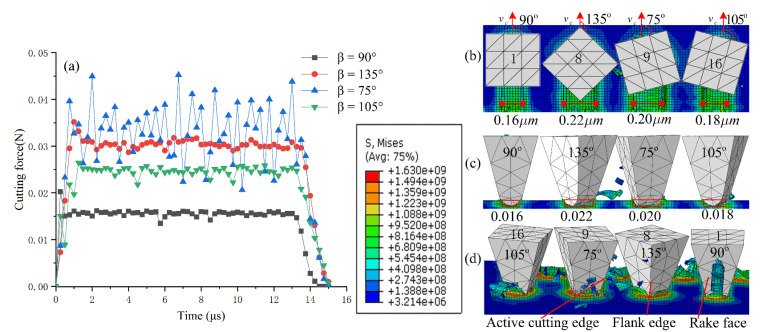
Effect of orientation angle on cutting force and material removal. (**a**) Honing force of different orientation angles; (**b**) Width of honing with different orientation angles; (**c**) Material removal rate of different orientation angles abrasive grains; (**d**) Influence of grain orientation angles on chip geometry.

Figure 10 shows the cutting forces and machining characteristics of abrasive grains 1, 8, 9 and 16 at different orientation angles under the process conditions of a honing depth 0.05 µm, contact trace length 30 µm and machining time of 15 μs. Evidently, with different orientation angles, the cutting force and the chip breakage effects are different. Grain 9 has the largest fluctuation in cutting force when its orientation angle is 70°. The main reason for this is that the major flank face and the secondary flank face of the grain are circular transition, which continuously crush the chips while cutting and produce fine chips, while the honing force constantly oscillates in the range of 0.02–0.045 N. For the other three orientation angles, the honing force of the abrasive grains is in the order of $F_{135{}^{\circ}}>F_{105{}^{\circ}}>F_{90{}^{\circ}}$. The honing force is more stable, but the chip breakage effect is poor.

As shown in Figure 10b,c, at an orientation angle 135°, the honing width of the abrasive grain is 0.22 µm, the average cross-sectional area of material removed by the abrasive grain is 0.011 µm^2^, the material removal rate is 0.022 µm^−3^/μs, and the force on the abrasive grain is the greatest. At the orientation angle 90°, the honing width of abrasive grains is 0.16 µm, the average cross-sectional area of material removed by abrasive grains is 0.008 µm^2^, the material removal rate is 0.016 µm^−3^/μs, and the honing force is the smallest. This shows that when the other process parameters and the geometry of abrasive grains remain unchanged, the magnitude of honing force for different orientation angles is positively related to honing width, average cross-sectional area, and material removal rate.

For grain 1, when the orientation is 90°, the elastoplastic deformation resistance and frictional resistance of the material mainly act on the rake face of the grain. The secondary cutting edge plays the major cutting role. When the material is removed, most of the material flows out from the rake face of the grain, and the chips are in the form of long strips, so the chip breaking effect is unsatisfactory. For grain 16, when the orientation angle is 105°, most of the chips are in the form of fine curls, flowing out from the rake face on the right side of the major cutting edge. For grain 8, when the orientation angle is 135°, the major cutting edge separates the interfering material into two parts, which flow out from the rake face and the major flank face, respectively. The chip flow has the combined effect of orientation angles 105° and 75°, with the best material removal efficiency. When the orientation angle of abrasive grain 9 is 75°, the elastic–plastic deformation resistance and the frictional resistance of the material mainly act on the major cutting edge and the flank face of the abrasive grain, and the material mainly flows out from the flank face. Since the transition between the flank face and the secondary flank face is circular, it facilitates chip breaking with fine, short flakes.

### 4.3. Relationship between the Number of Active Abrasive Grains and the Material Removal Rate

The material removal rate of the workpiece in the engagement zone is defined as the ratio of the actual material removal area to the theoretical material removal area. Let the residual area of the workpiece between abrasive grains after honing be *A_ij_*, and the material removal rate of active abrasive grains on the workpiece cross-section be expressed as:

(6) $$\eta_{N_{act}}^{’}=\frac{A-\sum_{i}^{j}A_{ij}}{A}\times 100\%\;$$

By determining the material removal rate of the active abrasive grains along the contact trail l, Equation (6) can be rewritten as:

(7) $$\eta_{N_{act}}=\frac{Al_{s,\Sigma }-\int_{0}^{l_{s,\Sigma }}A_{ij}dl_{s,\Sigma }}{Al_{s,\Sigma }}\times 100\%$$

where the residual area *A_ij_* is determined by the number of finite elements in the cutting area.

Given the process conditions of honing depth $T_{\mu d}=1\;\mu m$, cutting speed $v_{c}=8\;m/s$, contact trace length $l_{s,\Sigma }=30\;\mu m$, effective cutting width $b_{1}=32\;\mu m$, and theoretical removal area $A=30{\;\mu m}^{2}$, when the number of active abrasive grains randomly distributed in the cells of the engagement area are 17, 19 and 21, respectively, we can obtain the material removal rates $\eta_{17}=68.58\%$, $\eta_{19}=77.60\%$, $\eta_{21}=84.68\%$ by substituting the parameters into Equation (7). This indicates that, under a certain contact area, the higher the number of randomly distributed active abrasive grains, the smaller the residual area between abrasive grains after honing, and the higher the material removal efficiency.

## 5. Conclusions

In this work, an equivalent model of micro-edge honing with randomly distributed active abrasive grains was established. The effect of the random distribution states of active abrasive grains such as position, orientation and number on the material removal pattern, removal efficiency, honing force and surface quality of the workpiece were analyzed. Based on the obtained results, the following conclusions can be drawn:

(1) This paper simplified the honing process according to the experimental method of low-speed flat face grinding, and applied the finite-element method to visually simulate and explore the micro-edge cutting performance of active abrasive grains. The method is useful for simplifying complex cutting processes and studying the influence of specific machining conditions or parameters on the cutting performance, and can be employed in the study of machining characteristics of turning, milling, grinding, etc.
(2) Different distribution positions of active abrasive grains in the engagement area and different means of workpiece material removal give honing a combined processing characteristic of grinding, lapping and polishing. The active abrasive grains, at first contact with the workpiece, produce a high honing force, high material removal rate and poor surface roughness of the workpiece, while the abrasive grains that come into contact with the workpiece later show the opposite processing characteristics.
(3) Given the other process parameters remain unchanged, the honing forces of active abrasive grains for different orientation angles are mainly related to honing width, average cross-sectional area and material removal rate. The chip discharge direction and chip shape are different for different orientation angles. When the dislocation angle is 75°, the chips are small and discharge well, but the honing force is not stable.
(4) When more active abrasive grains are randomly distributed in the engagement area, the average interval distance between abrasive grains decreases, and less material remains after cutting, which is beneficial for improving the material removal efficiency.
(5) Limited by the accuracy of the experimental equipment and simulation capability, the information extraction and modeling of the active abrasive grains, the number of active abrasive grains, and the calculation of honing force are simplified. The error between the simulation results and the experimental results was within the acceptable range, which verified the reliability and accuracy of the simulation method.

## Figures and Tables

**Table 1:** Simulated processing parameters.

Parameters	Numerical Value
Number of CBN abrasives *N_d_*	15–21
Workpiece material	20CrMnTi
Cutting speed *v_c_*/(m/s)	0.1–16
Cutting depth *a_p_*/μm	0.12–3
Tool tip radius *ρ_s_*/μm	0.19–0.25
Cutting conditions	Dry cutting
Contact length *l_i_*/μm	30
Orientation angle *β*	75°, 90°, 105°, 135°

**Table 2:** Johnson–Cook plastic model constants for different materials.

Property	20CrMnTi	CBN
Young’s Modulus (GPa)	207	909
Poission’s ratio	0.25	0.12
Density (kg/mm^3^)	7.80 × 10^−6^	3.48 × 10^−6^
A (GPa)	3.03	-
B (GPa)	1.92	-
*n*	0.06	-
C	0.31	-
m	0.706	-

**Table 3:** Johnson-Cook damage constants for 20CrMnTi.

Property	*D*_1_	*D*_2_	*D*_3_	*D*_4_	*D*_5_	$T_{room}$	$T_{melt}$
20CrMnTi	−0.77	1.45	−0.47	0.014	3.87	20 ℃	1440 ℃

**Table 4:** Simulation and experiment parameters.

**Simulation**	Cutting speed $v_{c}\;\left(m/s\right)$	1.6
	Cutting depth $T_{\mu d}\;$(μm)	1.5
	Number of dynamic active grain $N_{dyn}$	17
	Cutting width $b_{1}\;$(μm)	32
	Effective contact length $l_{e,\Sigma }\;$(μm)	32
**Experiment**	Cutting speed $v_{c}\;\left(m/s\right)$	1.6
	Cutting depth $T_{\mu d}$ (μm)	1.5
	Gear module $M_{n}$	2.25
	Number of teeth for workpiece and honing wheel $z_{1}$, $z_{2}$	75,123
	Spiral angles for workpiece and honing wheel $\beta_{1}$, $\beta_{2}$	33°, 41.722°
	Pressure angle	41.722°

## Abbreviations

CBN	cubic boron nitride
CNC	computer numerical control
Σ	axis intersection angle
$N_{d}$	number of CBN abrasives
$N_{dyni}$	the dynamic active grains
$N_{act}$	the number of effective abrasive grains
$N_{dyn}$	the number of dynamic abrasive grains
$F_{ni}$	contact pressure
${\bar{F}}_{ng}$	the average honing force of a single abrasive grain
b	the tooth width
$b_{1}$	the effective cutting width
$l_{i}$	contact line/contact length
$l_{e,\Sigma }$	the effective contact length
$A_{ci}$	cross-sectional area
$T_{\mu i}$	depth of cut
J-C	Johnson–Cook
*a_p_*	cutting depth
*v_c_*	cutting speed
$v_{g}$	the sliding velocity
$v_{gev}$	the rolling velocity
$v_{gs}$	the spiral velocity
$\sigma_{p}$	the hydrostatic stress
$\sigma_{Mises}$	the von Mises stress
$\dot{\bar{\xi_{0}}}$	quasi-static compression test strain rate
$\dot{\bar{\xi }}$	equivalent plastic strain rate
*T*	the cutting temperature
$T_{room}$	the room temperature
$T_{melt}$	the melting temperature
$z_{1},\;z_{2}$	number of teeth
α	pressure angle
$M_{n}$	gear module
$\beta_{1},\;\beta_{2}$	Spiral angles
Simplified geometric model parameters of abrasive particles	
*l*	Length
*h*	height
$\rho_{s}$	tip radius
2θ	tip angle
b_2_	width
β	orientation angle
